# Targeting SREBP-dependent lipogenesis potentiates the anti-tumor activity of docetaxel by increasing membrane permeability and intracellular drug accumulation

**DOI:** 10.1038/s41388-025-03588-6

**Published:** 2025-10-04

**Authors:** Jiaqi Chen, Mu-En Wang, Alyssa R. Bawcom, Yi Lu, John M. Asara, Lei Li, Ming Chen

**Affiliations:** 1 Department of Pathology, Duke University School of Medicine, Duke Cancer Institute, Duke University, Durham, NC USA; 2 Department of Urology, The First Affiliated Hospital of Xi’an Jiaotong University, Xi’an, PR China; 3 Division of Signal Transduction, Beth Israel Deaconess Medical Center and Department of Medicine, Harvard Medical School, Boston, MA USA

**Keywords:** Prostate cancer, Cancer metabolism

## Abstract

Lipid metabolism is among the most frequently dysregulated metabolic processes in human cancer, yet how cellular lipids, the end products of lipogenesis, and their composition are altered to support various aspects of cancer remains poorly understood. Here, we show that targeting SREBP-dependent lipogenesis via FGH10019, an orally available SREBP inhibitor, enhances docetaxel-induced cytotoxicity in human prostate cancer cells in vitro and in vivo. Mechanistically, suppression of lipid biosynthesis leads to a shift in cellular lipid composition toward polyunsaturated lipids, resulting in increased membrane permeability and intracellular docetaxel accumulation. Thus, our findings reveal a critical role of de novo lipogenesis in protecting cancer cells from chemotherapeutics and suggest that treatment with lipogenesis inhibitors could improve the efficacy of chemotherapy against human prostate cancer.

## Introduction

Despite recent advances in therapeutic intervention, metastatic castration-resistant prostate cancer (mCRPC) remains an incurable disease. Since 2004, docetaxel has been the standard of care for patients with mCRPC [1], and it remains the only available life-prolonging therapy for mCRPC until 2010. Docetaxel is a semisynthetic taxane chemotherapeutic whose cytotoxic mechanism of action occurs through binding microtubules to prevent depolymerization, which arrests the cell cycle and eventually results in apoptosis [2]. Although treatment options for patients with mCRPC have vastly expanded in the past 15 years, docetaxel remains an essential treatment component for two reasons: first, the effects of novel androgen receptor signaling inhibitors (ARSI) are hampered by primary resistance in 30% of mCRPC cases [3], and second, cross-resistance is high during sequential treatment of novel ARSI, while chemotherapy is increasingly recognized as a therapy that potentially retains efficacy after the development of resistance to hormone therapies [3, 4]. As such, the identification of new strategies that sensitize tumor cells to docetaxel treatment is of critical importance and will have a clear clinical impact on prostate cancer patient care.

Metabolic reprogramming is a hallmark of cancer [5], and alterations in lipid metabolism comprise one well-documented aspect of this reprogramming [6]. Most lipids are synthesized from fatty acids (FAs), a family of molecules consisting of a terminal carboxyl group and a hydrocarbon chain of various carbon lengths and levels of saturation. In well-nourished individuals, de novo FA synthesis only minimally contributes to the lipid content of most adult tissues, as normal cells preferentially use exogenous FAs to satisfy their lipid requirements [7]; cancer cells, however, reactivate de novo FA synthesis irrespective of circulating lipid levels [8, 9], which indicates the crucial role played by FA synthesis in tumorigenesis. The expression of most enzymes involved in FA synthesis is controlled by the sterol regulatory element binding proteins (SREBPs) [10], which can be targeted via various chemical inhibitors, including FGH10019, an orally available small molecule that inhibits activation of SREBPs [11]. FAs play diverse roles at the level of the cell and the organism, from membrane biosynthesis and energy storage to signal transduction and post-translational modifications of proteins, to support cell proliferation, growth, and dissemination [6]. While the lipogenic phenotype in tumorigenesis is now widely recognized, how cellular lipids, the end products of lipogenesis, and their composition are altered to support various aspects of cancer is poorly understood.

To enter a cell, chemotherapeutic agents require interaction with its membrane, a barrier that can restrict the efficacy of chemotherapy [12, 13]. Membrane fluidity and permeability depend on the unsaturated/saturated lipid ratio. Saturated lipids favor ordered packing of the membrane as their straight hydrophobic tails interact with others through van der Waals interactions, while unsaturated lipids have at least one cis double bond that distorts the hydrophobic chain and prevents tight packing through steric hindrance. Thus, saturated lipids confer a more rigid and organized membrane and consequently decrease membrane fluidity and permeability. In contrast, unsaturated lipids decrease lipid packing in the membrane and improve membrane fluidity and permeability [14]. Notably, exogenous lipid analogs that increase membrane fluidity and permeability have been used in combinatorial therapies to enhance the uptake of various anti-tumor drugs and consequently maximize the anti-tumor activity of chemotherapeutic agents [12, 13], suggesting that combining lipid or membrane targeting strategies with available chemotherapy could represent a promising approach to improving the efficacy of chemotherapy [15].

Here, we demonstrate that targeting SREBP-dependent lipogenesis via FGH10019 enhances docetaxel-induced cytotoxicity through increased membrane permeability and intracellular docetaxel accumulation. Our results indicate that de novo lipogenesis protects cancer cells from chemotherapeutics, suggesting the therapeutic potential of lipogenesis inhibitors in improving the efficacy of chemotherapy in human prostate cancer.

## Materials and methods

### Plasmids, reagents, and antibodies

A membrane-tagged GFP plasmid (Lck-GFP) was purchased from Addgene (plasmid #61099). Docetaxel and FGH10019 were purchased from MedChemExpress. ANEP dyes (Di-4-ANEPPDHQ, D36802) and Hoechst 33342 (62249) were purchased from Thermo Fisher. The antibodies for Western blotting are listed below: anti-PARP (46D11) and anti-Cleaved PARP (7C9) antibodies were purchased from Cell Signaling Technology. Anti-HMGCS1(A304-590A) antibody was purchased from Bethyl Laboratories. Anti-IDI1 (NBP1-57587) was purchased from Novus Biologicals. Anti-HSP90 antibody (614019) was purchased from BD Bioscience.

### Cell culture and transfection

C4-2, 22Rv1, PC3, and DU145 cell lines were purchased from ATCC. Cells were checked for mycoplasma using the MycoAlert Mycoplasma Detection Kit (Lonza). The prostate cancer cells were cultured in RPMI basic medium supplemented with 10% fetal bovine serum, 2 mM glutamine, and 100 U/mL of penicillin-streptomycin (Gibco) and maintained at 37 °C with 5% CO_2_. Transfections were performed using Lipofectamine 2000 according to manufacturer’s instructions. In brief, 50 nM mixtures of two independent siRNA pairs targeting *SREBP*1 or *SREBP*2, or 1 μg of DNA plasmids, were transfected into 1 × 10^5^ cells in a 6-well dish. Cells were recovered into completed media after a 12-h transfection and then harvested for the downstream assays. Sequences of siRNA pairs were previously described [10].

### FRAP analyses

To assess membrane fluidity, C4-2 or PC-3 cells were seeded onto glass-bottom chamber slides (µ-Slide, Ibidi) in media supplemented with lipoprotein-deficient serum and transfected with a Lck-GFP plasmid (Addgene #61099) for 24 h. Following transfection, cells were treated for an additional 24 h with vehicle, 5 μM FGH10019, 1 nM docetaxel, or a combination of docetaxel and FGH10019. FRAP was conducted using a Zeiss 880 Airyscan Fast Inverted Confocal Microscope equipped with 488 nm laser. A defined region of interest (ROI) on the plasma membrane was photobleached with high-intensity laser exposure. Subsequent fluorescence recovery within the ROI was recorded over time using low laser power to minimize further photobleaching. The recovery kinetics were quantified by measuring fluorescence intensity within the ROI at each time point, and values were normalized to the pre-bleach intensity to assess membrane fluidity across treatment conditions.

### Analyses of the uptake of ANEP dyes and cell viability

Prostate cancer cells were cultured in media supplemented with lipoprotein-deficient serum for 2 days, then treated with vehicle, 5 μM FGH10019, 1 nM docetaxel, or a combination of docetaxel and FGH10019 for another 48 h followed by 1 μM ANEP dyes for 60 min. The cells were washed and lysed. The uptake of ANEP dyes was measured using a SpectraMax M3 Multi-Mode Microplate Reader (Molecular Devices) with an emission peak at 650 nM. Data were normalized using the intensity of Hoechst 33342 with an emission peak at 460 nM. Cell viability was measured using the CellTiter-Glo 3D Cell Viability Assay kit (Promega). Briefly, cells were plated in white-walled 96-well plates (Falcon) at the density of 1.0 × 10^4^ cells/well and treated with vehicle, 1 nM docetaxel, 5 μM FGH10019, or a combination of docetaxel and FGH10019 for 72 h. After the treatment, one volume of the CellTiter Glo reagent was added into each well, mixed on a thermomixer at 750 rpm for 5 min, and then incubated at room temperature for 20 min. The luminance signal for a 250 ms integration time was measured using a SpectraMax M3 Multi-Mode Microplate Reader (Molecular Devices).

### Western blotting

Cells were lysed in RIPA buffer (Boston BioProducts) supplemented with Complete Protease Inhibitor Cocktail (Roche) and Phosphatase Inhibitor Cocktail 2 (Sigma Aldrich). The protein concentration was quantified using the BCA protein assay kit (Thermo Scientific). Cell lysates were mixed with 6× Laemmli buffer (Boston BioProducts) and boiled at 95 °C for 5 min. Denatured proteins were separated on 10% SDS-PAGE and then transferred onto nitrocellulose membranes (GE Healthcare) using the standard wet transfer method. Membranes were blocked with 5% milk plus 0.1% Tween-20 in PBS for 1 h at room temperature, and then incubated with specific first antibodies overnight at 4 °C. The HRP-conjugated secondary antibodies and ECL substrate (GE Healthcare) were applied to visualize the bands of specific proteins.

### Quantification of membrane holes

Prostate cancer cells were treated with vehicle, 5 μM FGH10019, 1 nM docetaxel, or a combination of docetaxel and FGH10019 for 2 days. Cells were then dried with hexamethyldisilane and sputter-coated with 50–100 Å gold-palladium using a Denton Desk V Sputter Coater (Denton Vacuum, Moorestown, NJ). Visualization was performed with a FEI XL30 SEM-FEG (Thermo Fisher Scientific, MA) and operated at 5 kV by using InLens and TLD detection at a working distance of approximately 6.4 mm. Corresponding images over the cell bodies were acquired by SEM at ×2000, ×20,000, or ×65,000 magnification. TIFF images were captured at 645 × 522 pixels resolution with line averaging (a noise reduction algorithm). For quantitation of the number and size of holes in cell membrane, the high-magnification (×65,000) 8-bit images were imported into ImageJ version 1.52 (National Institutes of Health, Bethesda, MD). The level was set to 1 to enhance the visual contrast between the dark holes and the gray/white cell membrane. The images were then thresholded from 0 (lower limit) to 190–210 (upper limit), which filled in the holes. A 500 nm-radius circular ROI was drawn over the cell body in an area containing smooth holes that were biological, as opposed to jagged-edged holes that represented processing artifacts (reference). Within the 500 nm-radius circular ROI, the number of cellular membrane holes with size ≥100 nm^2^ were counted, and the average size of the holes within the ROI was calculated. The number and size of holes were then compared between different groups.

### Histology and IHC

Xenograft tumors were dissected and fixed in 4% paraformaldehyde for histology and IHC analysis. For staining, the tissues were embedded in paraffin according to standard procedures. 5 μm sections were cut and processed for histology or immunostaining. The following primary antibodies were used for Ki67 (Thermo Fisher Scientific, SP6, 1:100) and cleaved PARP (Cell Signaling Technology, 7C9, 1:400). The stained slides were visualized by a bright-field microscope. Quantification of Ki-67 and cleaved PARP staining was performed with NIH ImageJ.

### Lipidomics by untargeted high-resolution liquid chromatography–tandem mass spectrometry (LC–MS/MS) and measurement of docetaxel by high-performance liquid chromatography

Lipidomic analysis and measurement of docetaxel were performed and quantified as previously described in references [10, 16] and [17], respectively. Briefly, prostate cancer cells in 10 cm dishes were treated with 1 nM docetaxel in the absence or presence of 5 μM FGH10019 for 48 h. Cell pellets were then harvested in PBS, and protein content was measured using a BCA protein assay kit (Thermo Scientific) for sample normalization. For the lipidomic analysis, non-polar lipids were extracted with MTBE and dried using a SpeedVac Vacuum Concentrator (Thermo Scientific) with no heat. Lipid samples were resuspended in 35 μl of 50% isopropanol (IPA)/50% MeOH. Ten microliters of samples were injected for reversed-phase (C_18_) LC–MS/MS using a hybrid QExactive Plus Orbitrap mass spectrometer (Thermo Fisher Scientific) coupled to an Agilent 1100 HPLC in DDA mode using positive/negative ion polarity switching (Top 8 in both modes). For the measurement of docetaxel, polar metabolites, including docetaxel, were extracted with 80% methanol (−80 °C) for 20 min. Dried metabolite pellets were re-suspended in 20 μL LC/MS grade water, 5 μL were injected over a 15 min gradient using a 5500 QTRAP triple quadrupole mass spectrometer (AB/SCIEX) coupled to a Prominence UFLC HPLC system (Shimadzu) via SRM of a total of 287 SRM transitions using positive/negative polarity switching corresponding to 258 unique endogenous water-soluble metabolites. A series of concentrations of docetaxel from below and above the expected quantities were provided to make a concentration curve for absolute quantification.

### Cell-line derived xenografts and in vivo treatment

1 × 10^6^ PC3 or C4-2 cells were mixed with 100 μL of Matrigel (Corning) and implanted subcutaneously into the right flank of 6–8 weeks old male nude mice (Foxn1nu, Jackson Laboratory). The tumor volume was measured using calipers and calculated as L × W^2^ × 0.52, where the L (length) stands for the largest tumor diameter and the W (width) stands for the diameter perpendicular to the length. When tumor volumes reached ~80–100 mm^3^, mice were randomly allocated into four groups and assigned one of four treatment regimens: vehicle, docetaxel (i.p., 4 mg/kg, dissolved in 10% DMSO/40% PBS/50% PEG-400, twice a week), FGH10019 (oral gavage, 20 mg/kg, dissolved in 10% DMSO/90% PEG-400, three time a week) or a combination of docetaxel with FGH10019.

### Statistical analysis

No statistics was applied to determine sample size. The studies involving mice were randomized. The investigators were not blinded to allocation during experiments and outcome assessment. No animals were excluded from the analysis. For analysis of average data, datasets were compared using unpaired two-tailed Student’s *t* tests. For analysis of the synergistic effect of docetaxel and FGH10019, datasets were compared using two-way ANOVA with Bonferroni’s post-hoc tests to assess their interaction term. For analysis of the FRAP recovery curves, the data were fitted to a one-phase association model. To compare the recovery dynamics between treatment groups, global and individual curve fittings were performed, and statistical significance was assessed using the extra sum-of-squares F test. *P* values of <0.05 were considered statistically significant. Statistical tests were executed using GraphPad Prism software.

## Results

### Inhibition of SREBP-dependent lipogenesis decreases levels of total cellular lipids and lipid saturation

**Fig. 1:**
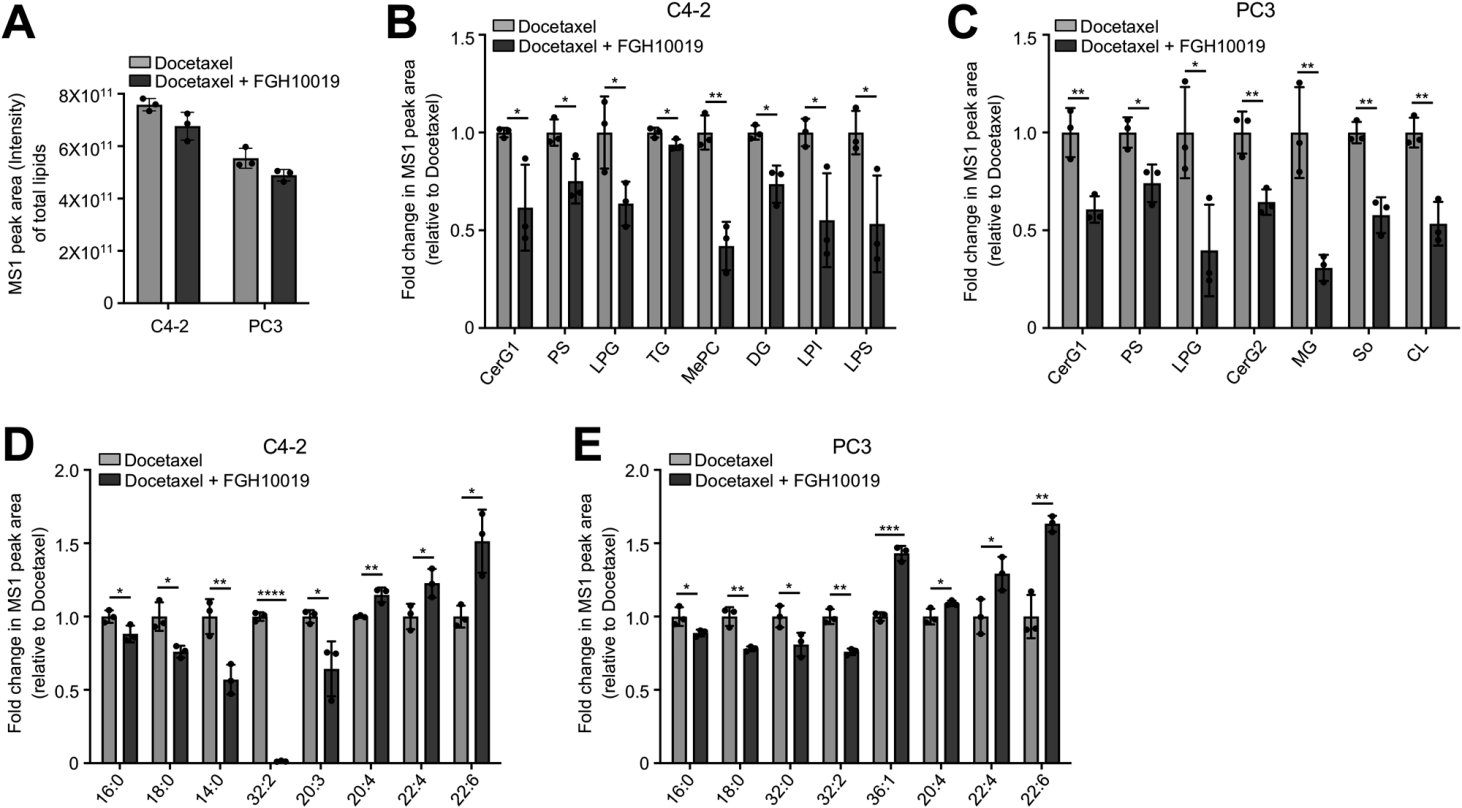
Inhibition of SREBP-dependent lipogenesis decreases levels of total cellular lipids and lipid saturation. **A** Bar graph showing the relative MS1 peak area intensity of all identifiable lipids in C4-2 or PC3 cells treated with 1 nM docetaxel in the absence or presence of 5 μM FGH10019 for 48 h. **B**−**E** A statistically significant alteration in abundance of various lipid classes (**B**, **C**) or fatty acyl chains (**D**, **E**) in C4-2 (**B**, **D**) or PC3 (**C**, **E**) cells treated with 1 nM docetaxel in the absence or presence of 5 μM FGH10019 for 48 h. MS1 peak area represents the relative quantification of the intensity of identified lipids across the sample set. CerG1 simple Glc series 1, CerG2 simple Glc series 2, PS phosphatidylserine, LPG lysophosphatidylglycerol, TG triglyceride, MePC monoetherphosphatidylcholin, DG diglyceride, LPI lysophosphatidylinositol, LPS lysophosphatidylserine, MG monoglyceride, So sphingosine, CL cardiolipin. Unpaired two-tailed *t* test was used to determine significance. *P < 0.05, **P < 0.01, ***P < 0.001, ****P < 0.0001. All data are mean ± s.d. from *n* = 3 biological replicates.

Plasma membrane fluidity and permeability dictate the entry of chemotherapeutic agents into cells and are affected by lipid saturation levels [12–14]. Previous reports have shown that activation of de novo lipogenesis increases lipid saturation [10, 18], and we therefore assessed whether inhibition of SREBP-dependent lipogenesis by FGH10019, an orally available SREBP inhibitor [11], could modulate lipid composition, cell membrane dynamics, and uptake of docetaxel in human prostate cancer cells. To this end, we performed a global lipidomic analysis in docetaxel-treated C4-2 and PC3 cells in the absence or presence of FGH10019 using untargeted high-resolution liquid chromatography–tandem mass spectrometry (LC–MS/MS). These analyses identified 1622 lipid ions in human prostate cancer cell lines (Tables S1 and S2), which belonged to 36 classes of lipids (Table S3). Within all the identified lipid ions, 415 distinct fatty acyl chains were found, varying in chain length from 4 to 48 carbons and in double bonds from 0 to 12 (Table S4). These results were largely similar to our previous findings in both human and murine prostate cancer cells and tissues [10, 16], and as expected, FGH10019 treatment lowered levels of total lipids in both C4-2 and PC3 cells (Fig. 1A). 27 out of 36 lipid classes identified showed decreased abundance in FGH10019-treated C4-2 cells (Fig. S1A and Table S3), and the decreases in 8 of these lipid classes were statistically significant (Figs. 1B and S1A and Table S3). In FGH10019-treated PC3 cells, 29 out of 35 identified lipid classes showed decreased abundance (Fig. S1B and Table S3), and of these the decreases in 7 lipid classes were statistically significant (Figs. 1C, S1B and Table S3).

We subsequently examined the impact of inhibition of de novo lipogenesis on the levels of lipid saturation. Among 415 fatty acyl chains identified (Table S4), we compared the levels of the 30 most abundant fatty acyl chains to assess the alterations in lipid saturation in docetaxel-treated prostate cancer cells in the absence or presence of FGH10019 (Fig. S1C, D). A total of eight fatty acyl chains had significant alterations upon FGH10019 treatment in both C4-2 and PC3 cells (Fig. 1D, E). While FGH10019 treatment significantly reduced the levels of saturated palmitoyl (16:0) and stearoyl (18:0) fatty acyl chains, two main end-products of de novo lipogenesis, it also led to significantly increased abundance in polyunsaturated fatty acyl chains (20:4, 22:4, and 22:6) in both C4-2 and PC3 cells (Fig. 1D, E). These results suggest that suppression of lipid biosynthesis leads to a shift in cellular lipid composition toward polyunsaturated lipids and decreases levels of lipid saturation.

### Inhibition of SREBP-dependent lipogenesis increases cell membrane dynamics and uptake of docetaxel

**Fig. 2:**
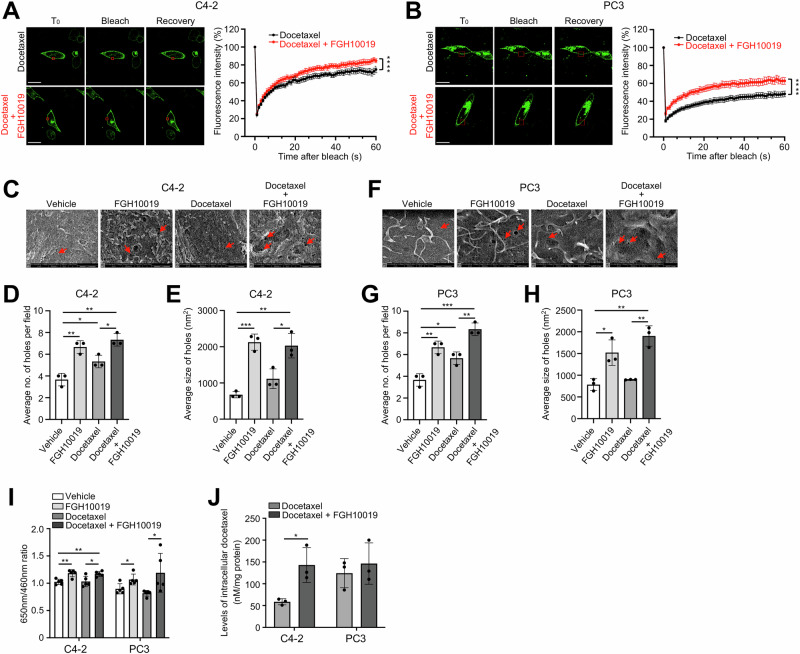
Inhibition of SREBP-dependent lipogenesis increases cell membrane dynamics and the uptake of docetaxel. **A**, **B** Representative images and quantitation of FRAP analysis of C4-2 (**A**) or PC3 (**B**) cells. Prostate cancer cells were transfected with a Lck-GFP plasmid for 24 h and then treated with 1 nM docetaxel in the absence or presence of 5 μM FGH10019 for another 24 h. Fluorescence was bleached in a specific region (red rectangle in **A**, **B**). Fluorescence recovery was then analyzed at the indicated time points. Scale bar, 20 μm. **C**−**H** Representative images and quantitation of the number and size of plasma membrane holes between C4-2 (**C**−**E**) or PC3 (**F**−**H**) cells treated with vehicle, 5 μM FGH10019, 1 nM docetaxel, or a combination of docetaxel and FGH10019 for 48 h. Arrows in **C** and **F** indicate plasma membrane holes. **I** Comparison of cellular uptake of ANEP dyes between C4-2 or PC3 cells treated with vehicle, 5 μM FGH10019, 1 nM docetaxel, or a combination of docetaxel and FGH10019 for 48 h. The intensity of ANEP dyes was detected with an emission peak at 650 nM with a normalization using Hoechst 33342. **J** Comparison of docetaxel concentration between C4-2 or PC3 cells treated with 1 nM docetaxel in the absence or presence of 5 μM FGH10019 for 48 h. The quantification of docetaxel concentration was normalized by protein content in treated cells. In **A**, **B**, statistical significance was assessed using the extra sum-of-squares F test. In **D**−**J**, unpaired two-tailed *t* test was used to determine significance. *P < 0.05, **P < 0.01, ***P < 0.001, ****P < 0.0001. All data are mean ± s.d. from *n* = 3 – 5 biological replicates.

Given the potential link between lipid composition and cell membrane dynamics [12–14], we next assessed whether modulation of de novo lipogenesis could also affect membrane fluidity and permeability. We first investigated the effect of FGH10019 on fluorescence recovery after photobleaching (FRAP) in C4-2 and PC3 cell lines. Briefly, prostate cancer cells were cultured in medium supplemented with 10% lipoprotein-deficient serum to limit the availability of exogenous lipids, transfected with a membrane-tagged GFP plasmid, and subjected to treatment with either vehicle or FGH10019 in the absence or presence of docetaxel. We then bleached the fluorescence in a small area of cell membrane and compared the recovery of fluorescence between vehicle- and FGH10019-treated cells. We found that inhibition of lipogenesis by FGH10019 led to a faster fluorescence recovery rate in C4-2 cells both in the absence (Fig. S2A) and presence of docetaxel treatment (Fig. 2A). Similar results were obtained in PC3 cells (Figs. 2B and S2B). To further demonstrate the effect of modulation of de novo lipogenesis on cell membrane dynamics, we utilized scanning electron microscopy to compare the number and size of plasma membrane holes in prostate cancer cells treated with vehicle or FGH10019 in the absence or presence of docetaxel, and found that FGH10019 treatment significantly increased the number and size of plasma membrane holes both in the absence and presence of docetaxel treatment in C4-2 (Fig. 2C–E) and PC3 cells (Fig. 2F–H). These findings are consistent with earlier studies showing that elevated levels of unsaturated lipids improve membrane fluidity and permeability [14].

Because increased membrane permeability is known to promote the uptake of chemotherapeutics [12–14], we then proceeded to determine whether inhibition of lipid biosynthesis is associated with higher cellular uptake of docetaxel. Using the same cell line samples analyzed in Fig. 2C–H, we compared the rate of uptake of ANEP dyes (Di-4-ANEPPDHQ) between vehicle- and FGH10019-treated cells and found that the rate of cellular uptake of ANEP dyes was higher in FGH10019-treated C4-2 or PC3 cells both in the absence and presence of docetaxel (Fig. 2I). Finally, we measured the levels of intracellular docetaxel through high performance lipid chromatography (HPLC) in docetaxel-treated prostate cancer cells in the absence or presence of co-treatment with FGH10019 and found that levels of intracellular docetaxel were significantly higher in C4-2 cells co-treated with FGH10019 and docetaxel (Fig. 2J). A trend of increasing levels of intracellular docetaxel was also observed in PC3 cells co-treated with FGH10019 and docetaxel (Fig. 2J), although this was not statistically significant, possibly due to high basal uptake levels of docetaxel (Fig. 2J). These data indicate that targeting de novo lipogenesis results in increased number and size of plasma membrane holes and subsequent higher cellular uptake of docetaxel.

### Targeting SREBP-dependent lipogenesis potentiates the anti-tumor activity of docetaxel in prostate cancer both in vitro and in vivo

**Fig. 3:**
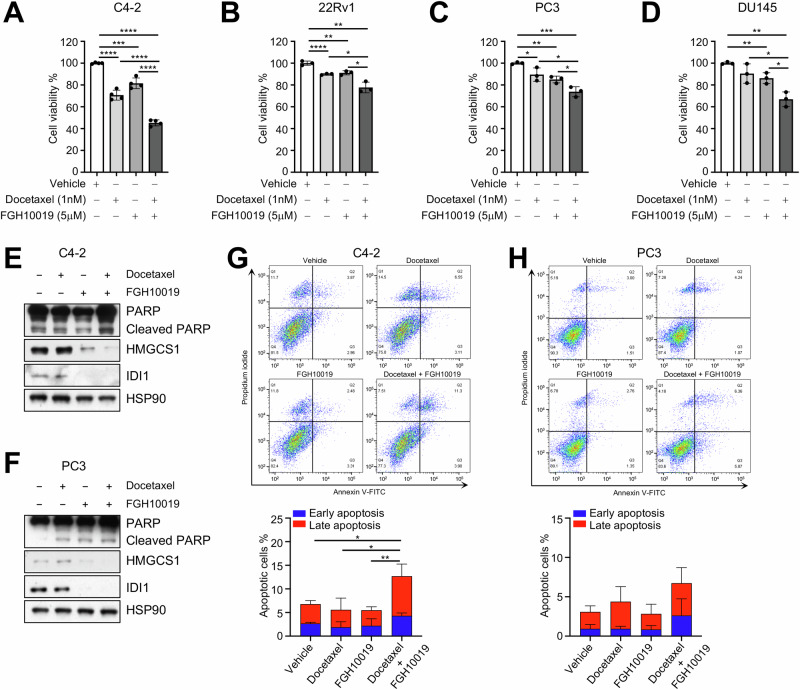
Targeting SREBP-dependent lipogenesis potentiates the anti-tumor activity of docetaxel in prostate cancer in vitro. **A**–**D** 72-h cell viability assay of C4-2 (**A**), 22RV1 (**B**), PC3 (**C**), or DU145 (**D**) cells treated with vehicle, 1 nM docetaxel, 5 μM FGH10019, or a combination of docetaxel and FGH10019. **E**, **F** Immunoblot (IB) analysis of lysates from C4-2 (**E**) or PC3 (**F**) cell line treated with vehicle, 1 nM docetaxel, 5 μM FGH10019, or a combination of docetaxel and FGH10019 for 72 h. **G**, **H** Representative fluorescence-activated cell sorting (FACS) plots and quantitation of early and late apoptotic cells from C4-2 (**G**) or PC3 (**H**) cell line treated with vehicle, 1 nM docetaxel, 5 μM FGH10019, or a combination of docetaxel and FGH10019 for 72 h. In **A**–**D**, **G**, **H**, unpaired two-tailed *t* test was used to determine significance. *P < 0.05, **P < 0.01, ***P < 0.001, ****P < 0.0001. All data are mean ± s.d. from *n* = 3–5 biological replicates.

We next evaluated the consequence of increased uptake of docetaxel in prostate cancer cells cotreated with docetaxel and FGH10019. Docetaxel in the nM ranges can exhibit cytotoxicity toward various prostate cancer cell lines [19–21]. We examined the inhibitory effects of FGH10019 as a single agent on several AR-positive and -negative prostate cancer cell lines. IC_50_ values of FGH10019 for prostate cancer cell lines ranged from 9 to 22 μM **(**Fig. S3A**)**. We next evaluated the combined inhibitory effects of docetaxel and FGH10019 on prostate cancer cell lines at their suboptimal doses and observed that co-treatment with both agents led to significantly additive suppression on cell growth in all prostate cancer cell lines tested (Fig. 3A–D). As expected, inhibition of SREBP by FGH10019 induced lower expression of HMG-CoA synthase 1 (HMGCS1) and isopentenyl-diphosphate delta isomerase 1 (IDI1), two well-known SREBP-regulated enzymes that participate in lipid biosynthesis [22] (Figs. 3E, F and S3B, C), while co-treatment with docetaxel and FGH10019 enhanced the induction of apoptosis, as indicated by higher expression of cleaved PARP (Figs. 3E, F and S3B, C) and higher percentages of early and late apoptotic cells (Figs. 3G, H and S3D, E). In line with these results, knockdown of *SREBP-*1*/*2 via small interfering RNAs (siRNAs) sensitized prostate cancer cells C4-2 and PC3 to docetaxel-induced cytotoxicity (Fig. S3F). These results suggest that targeting SREBP-dependent lipogenesis results in higher cellular uptake of docetaxel, which would ultimately lead to elevated levels of docetaxel-induced cell death.

**Fig. 4:**
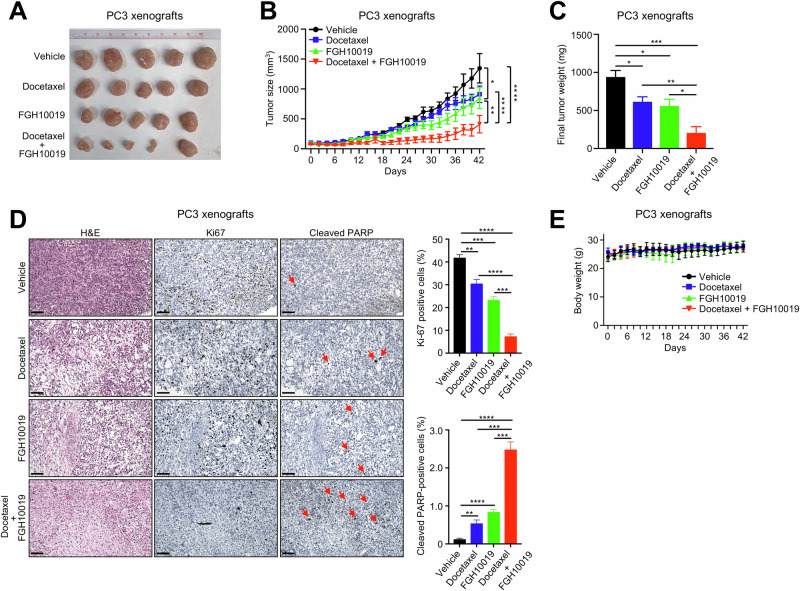
Targeting SREBP-dependent lipogenesis potentiates the anti-tumor activity of docetaxel in prostate cancer in vivo. **A**–**C** Tumor images (**A**), tumor volume over the course of treatment (**B**), and final tumor weight (**C**) of PC3 xenografts after 6-week treatment with vehicle, docetaxel, FGH10019, or a combination of docetaxel with FGH10019. n = 5 mice per group. **D** H&E, IHC, and quantification of Ki-67 and cleaved PARP staining of PC3 xenografts from different treatment regimens. Arrows indicate apoptotic cells. Scale bars, 50 μm. **E** Body weight of nude mice from different treatment regimens implanted with PC3 xenografts. Docetaxel was administered i.p. at a dose of 4 mg/kg body weight twice per week. FGH10019 was administered orally at a dose of 20 mg/kg body weight three times per week. In **B**, two-way ANOVA with Tukey’s multiple-comparison test with Bonferroni’s post-hoc tests was used to determine significance. In **C**, **D** unpaired two-tailed *t* test was used to determine significance. *P < 0.05, **P < 0.01, ***P < 0.001, ****P < 0.0001. All data are mean ± s.d.

To further test whether targeting SREBP-dependent lipogenesis would enhance the cytotoxicity of docetaxel in vivo, we carried out preclinical studies of FGH10019 in combination with docetaxel against prostate cancer growth using cell line-derived xenograft (CDX) models. In a PC3 CDX model of prostate cancer, treatment with either docetaxel or FGH10019 for 6 weeks suppressed the growth of PC3 xenografts, while cotreatment with docetaxel and FGH10019 led to strong synergistic anti-tumor activity and further suppressed the growth of PC3 xenografts to the greatest degree (Fig. 4A−C). Consistently, PC3 xenograft tumors from the combined treatment regimen displayed a drastic decrease in the frequency of mitotic cells positive for Ki-67 staining, along with a concomitantly enhanced induction of apoptosis, as indicated by higher expression of cleaved PARP (Fig. 4D). Importantly, none of the four treatment regimens showed obvious toxicity in mice as the treatment did not alter body weight (Fig. 4E). In another C4-2 CDX model of prostate cancer, we found that treatment with either docetaxel or FGH10019 for 3 weeks had no significant effect on the growth of C4-2 xenografts, while co-treatment with FGH10019 and docetaxel significantly inhibited the growth of C4-2 xenografts (Fig. S3G, H). Taken together, these preclinical data indicate that targeting SREBP-dependent lipogenesis potentiates the anti-tumor activity of docetaxel against prostate cancer both in vitro and in vivo.

## Discussion

Aberrant lipid metabolism is now widely recognized as a key element in promoting the aggressiveness of cancer, and its related genes and pathways have been identified as potential new targets for cancer therapy [6]. However, the metabolic plasticity which enables cancer cells to switch between FA synthesis, FA uptake, and degradation to meet their needs for nutrients poses significant challenges to targeting lipid metabolism by means of single agents [23]. Successful therapies targeting lipid metabolism may therefore depend on a better understanding of the tumor-promoting activity associated with specific types of cancer, which may require combinatorial treatment to potentiate anti-tumor activity [6].

Docetaxel is one of the cornerstones of chemotherapy for prostate cancer [1], but there is a critical need to improve its efficacy against advanced prostate cancer by maximizing its anti-tumor activity. Docetaxel crosses the plasma membrane through passive diffusion, and thus plasma membrane permeability is a major factor influencing its efficacy [24]. Our data reveal that lipid metabolism may inhibit the response to docetaxel therapy by increasing the levels of saturated FAs, thus forming packed lipid membranes that limit the entry of docetaxel into prostate cancer cells. We show that inhibition of SREBP-dependent lipogenesis by FGH10019 leads to a shift in cellular lipid composition toward polyunsaturated lipids (Fig. 1D, E), presumably due to a selective reduction in the levels of saturated FAs; as mammalian cells have a limited ability to synthesize polyunsaturated FAs due to their lack of delta-12 and -15 desaturases [25], their levels of polyunsaturated FAs are less impacted by inhibition of de novo lipogenesis. A skew of the lipid ratio toward polyunsaturated FAs in turn results in increased membrane fluidity (Figs. 2A, B, and S2A, B), permeability (Fig. 2C−H), and intracellular docetaxel accumulation (Fig. 2J). Importantly, in all prostate cell lines tested, inhibition of cell viability was potentiated by combinatorial treatment with docetaxel and FGH10019 (Fig. 3A−D). This additive effect is presumably due to increased cellular uptake of docetaxel, which triggers heightened apoptosis (Fig. 3E−H); this is further corroborated by preclinical studies of FGH10019 and docetaxel in CDX models of prostate cancer (Figs. 4 and S3G, H). Notably, this combinatorial treatment is effective in prostate cancer cells regardless of *AR* status. Further studies are needed to determine whether inhibition of SREBP has a similar impact on the uptake and cytotoxicity of other chemotherapeutics in cancer cell lines of various histological origins. Furthermore, given the crucial role of lipid metabolism in various aspects of cancer development [6], it remains possible that other molecular changes induced by SREBP blockade may contribute to potentiation of the anti-tumor activity of docetaxel. For example, one effect of the inhibition of SREBP is the induction of ER-stress, which leads to inhibition of protein synthesis through increased phosphorylation of eIF2α [26].

The SREBP signature is highly enriched in tumor samples from mCRPC-affected individuals [10], a patient population in which docetaxel chemotherapy has gained clinical traction, suggesting that combinatorial therapy with FGH10019 and docetaxel is relevant and promising in the treatment of advanced prostate cancer. Notably, our data indicate that this combinatorial therapy displays no obvious toxicity in mice (Fig. 4C), demonstrating its feasibility as a cancer therapy. As a small chemical SREBP inhibitor, the pharmacokinetics of FGH10019 in vivo have yet to be optimized [11]. Further preclinical studies are needed to evaluate whether systematic optimization of dose and duration of FGH10019 will yield improved therapeutic potential in combination with docetaxel treatment.

Together, our findings reveal that de novo lipogenesis protects cancer cells from chemotherapeutics, suggesting that treatment with SREBP inhibitors may potentially improve the efficacy of chemotherapy against human prostate cancer.

## Supplementary information

Supplementary Figures 1–3

Supplementary Tables 1–4

## Data Availability

The authors declare that the data supporting the findings of this study are available within the article and its Supplementary Information files. All relevant data are available from the authors upon request.

## References

[1] Tannock IF, de Wit R, Berry WR, Horti J, Pluzanska A, Chi KN, et al. Docetaxel plus prednisone or mitoxantrone plus prednisone for advanced prostate cancer. N Engl J Med. 2004;351:1502–12. 15470213 10.1056/NEJMoa040720

[2] Yvon AM, Wadsworth P, Jordan MA. Taxol suppresses dynamics of individual microtubules in living human tumor cells. Mol Biol Cell. 1999;10:947–59. 10198049 10.1091/mbc.10.4.947 PMC25218

[3] Mottet N. Metastatic castration-resistant prostate cancer: major progress leading to even more questions. Eur Urol Focus. 2016;2:562–4. 28723523 10.1016/j.euf.2016.10.006

[4] Sartor O, de Bono JS. Metastatic prostate cancer. N Engl J Med. 2018;378:645–57. 29412780 10.1056/NEJMra1701695

[5] Hanahan D, Weinberg RA. Hallmarks of cancer: the next generation. Cell. 2011;144:646–74. 21376230 10.1016/j.cell.2011.02.013

[6] Chen M, Huang J. The expanded role of fatty acid metabolism in cancer: new aspects and targets. Precis Clin Med. 2019;2:183–91. 31598388 10.1093/pcmedi/pbz017 PMC6770278

[7] Weiss L, Hoffmann GE, Schreiber R, Andres H, Fuchs E, Körber E, et al. Fatty-acid biosynthesis in man, a pathway of minor importance. Purification, optim assay cond, organ distrib fat-acid synthase. Biol Chem Hoppe Seyler. 1986;367:905–12. 3790257 10.1515/bchm3.1986.367.2.905

[8] Sabine JR, Abraham S, Chaikoff IL. Control of lipid metabolism in hepatomas: insensitivity of rate of fatty acid and cholesterol synthesis by mouse hepatoma BW7756 to fasting and to feedback control. Cancer Res. 1967;27:793–9. 4290628

[9] Ookhtens M, Kannan R, Lyon I, Baker N. Liver and adipose tissue contributions to newly formed fatty acids in an ascites tumor. Am J Physiol. 1984;247:R146–153. 6742224 10.1152/ajpregu.1984.247.1.R146

[10] Chen M, Zhang J, Sampieri K, Clohessy JG, Mendez L, Gonzalez-Billalabeitia E, et al. An aberrant SREBP-dependent lipogenic program promotes metastatic prostate cancer. Nat Genet. 2018;50:206–18. 29335545 10.1038/s41588-017-0027-2 PMC6714980

[11] Kamisuki S, Shirakawa T, Kugimiya A, Abu-Elheiga L, Choo HY, Yamada K, et al. Synthesis and evaluation of diarylthiazole derivatives that inhibit activation of sterol regulatory element-binding proteins. J Med Chem. 2011;54:4923–7. 21561152 10.1021/jm200304y PMC3136361

[12] Veldman RJ, Zerp S, van Blitterswijk WJ, Verheij M. N-hexanoyl-sphingomyelin potentiates in vitro doxorubicin cytotoxicity by enhancing its cellular influx. Br J Cancer. 2004;90:917–25. 14970874 10.1038/sj.bjc.6601581 PMC2410169

[13] van Hell AJ, Melo MN, van Blitterswijk WJ, Gueth DM, Braumuller TM, Pedrosa LR, et al. Defined lipid analogues induce transient channels to facilitate drug-membrane traversal and circumvent cancer therapy resistance. Sci Rep. 2013;3:1949. 23739489 10.1038/srep01949 PMC3674426

[14] Golden GM, McKie JE, Potts RO. Role of stratum corneum lipid fluidity in transdermal drug flux. J Pharm Sci. 1987;76:25–28. 3585718 10.1002/jps.2600760108

[15] Zalba S, Ten Hagen TL. Cell membrane modulation as adjuvant in cancer therapy. Cancer Treat Rev. 2017;52:48–57. 27889637 10.1016/j.ctrv.2016.10.008 PMC5195909

[16] Wang ME, Chen J, Lu Y, Bawcom AR, Wu J, Ou J, et al. RB1-deficient prostate tumor growth and metastasis are vulnerable to ferroptosis induction via the E2F/ACSL4 axis. J Clin Investig. 2023;133:e166647. 10.1172/JCI166647 PMC10178842 36928314

[17] Loh ZN, Wang ME, Wan C, Asara JM, Ji Z, Chen M. Nuclear PTEN regulates thymidylate biosynthesis in human prostate cancer cell lines. Metabolites. 2023;13:939. 10.3390/metabo13080939 PMC10456368 37623882

[18] Rysman E, Brusselmans K, Scheys K, Timmermans L, Derua R, Munck S, et al. De novo lipogenesis protects cancer cells from free radicals and chemotherapeutics by promoting membrane lipid saturation. Cancer Res. 2010;70:8117–26. 20876798 10.1158/0008-5472.CAN-09-3871

[19] Yang C, Zhang W, Wang J, Chen P, Jin J. Effect of docetaxel on the regulation of proliferation and apoptosis of human prostate cancer cells. Mol Med Rep.2019;19:3864–70. 30864701 10.3892/mmr.2019.9998

[20] Guan W, Hu J, Yang L, Tan P, Tang Z, West BL, et al. Inhibition of TAMs improves the response to docetaxel in castration-resistant prostate cancer. Endocr Relat Cancer. 2019;26:131–40. 30400004 10.1530/ERC-18-0284 PMC6226051

[21] Domingo-Domenech J, Vidal SJ, Rodriguez-Bravo V, Castillo-Martin M, Quinn SA, Rodriguez-Barrueco R, et al. Suppression of acquired docetaxel resistance in prostate cancer through depletion of notch- and hedgehog-dependent tumor-initiating cells. Cancer Cell. 2012;22:373–88. 22975379 10.1016/j.ccr.2012.07.016 PMC5989708

[22] Horton JD, Shah NA, Warrington JA, Anderson NN, Park SW, Brown MS, et al. Combined analysis of oligonucleotide microarray data from transgenic and knockout mice identifies direct SREBP target genes. Proc Natl Acad Sci USA. 2003;100:12027–32. 14512514 10.1073/pnas.1534923100 PMC218707

[23] Vishwa R, BharathwajChetty B, Girisa S, Aswani BS, Alqahtani MS, Abbas M, et al. Lipid metabolism and its implications in tumor cell plasticity and drug resistance: what we learned thus far? Cancer Metastasis Rev. 2024;43:293–319. 38438800 10.1007/s10555-024-10170-1

[24] Bourgaux C, Couvreur P. Interactions of anticancer drugs with biomembranes: what can we learn from model membranes? J Control Release. 2014;190:127–38. 24859379 10.1016/j.jconrel.2014.05.012

[25] Watts JL. Fat synthesis and adiposity regulation in Caenorhabditis elegans. Trends Endocrinol Metab. 2009;20:58–65. 19181539 10.1016/j.tem.2008.11.002 PMC2665873

[26] Griffiths B, Lewis CA, Bensaad K, Ros S, Zhang Q, Ferber EC, et al. Sterol regulatory element binding protein-dependent regulation of lipid synthesis supports cell survival and tumor growth. Cancer Metab. 2013;1:3. 24280005 10.1186/2049-3002-1-3 PMC3835903

