# Supplementary material for: Targeting SREBP-dependent lipogenesis potentiates the anti-tumor activity of docetaxel by increasing membrane permeability and intracellular drug accumulation
Source: Oncogene. 2025 Oct 4;44(45):4405–12. doi: 10.1038/s41388-025-03588-6 (PMC12583198; doi:10.1038/s41388-025-03588-6)
Supplement: Supplementary file 1 — Supplementary Figures 1–3 [file 41388_2025_3588_MOESM1_ESM.pdf]

Supplementary Information

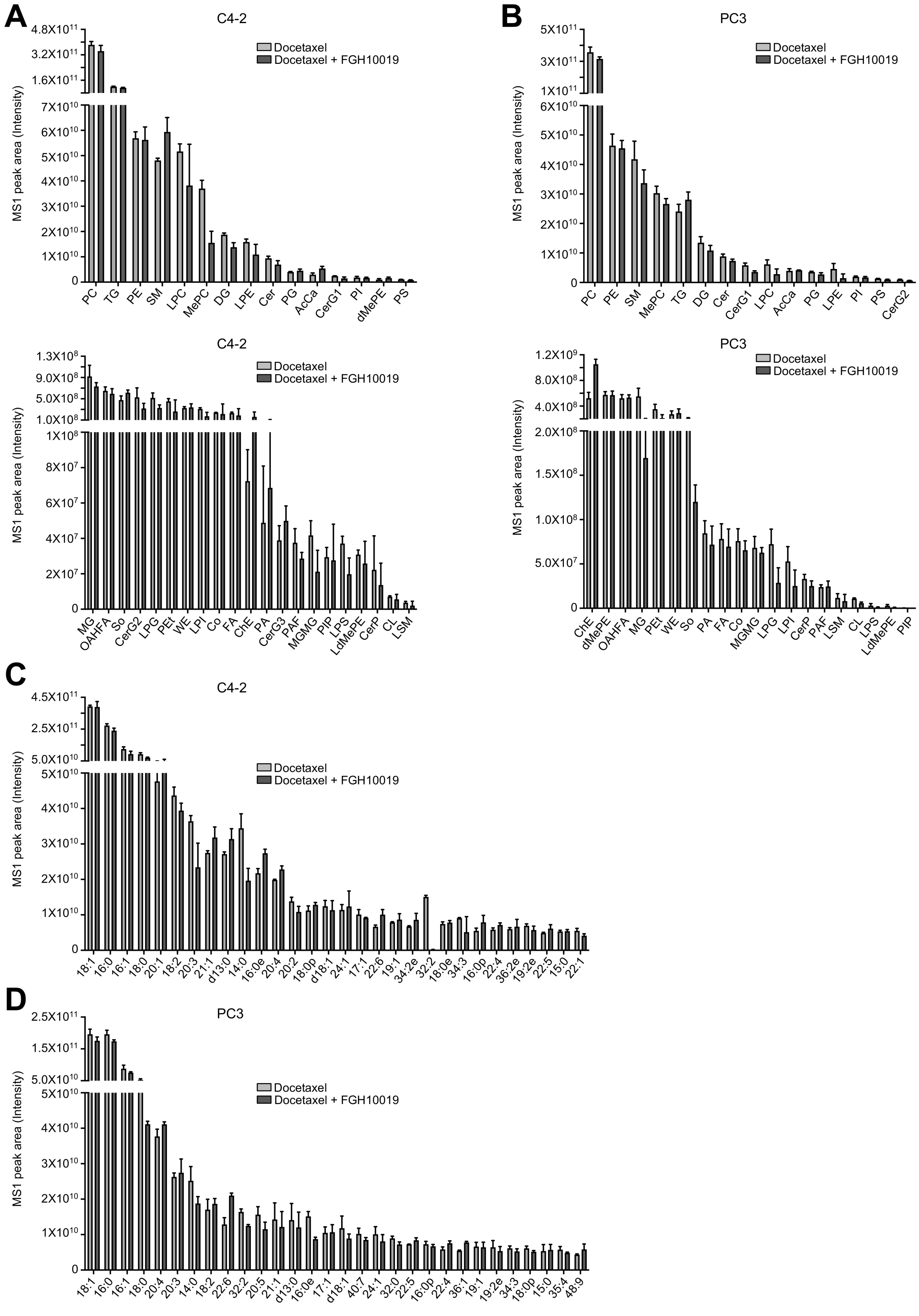

Fig. S1. Inhibition of SREBP-dependent lipogenesis decreases the levels of total cellular lipids and lipid saturation. A–D The relative intensity of all the identifiable 36 lipid classes (**A**, **B**) or the 30 most abundant fatty acyl chains (**C**, **D**) in C4-2 (**A**, **C**) or PC3 (**B**, **D**) cells treated with 1nM docetaxel in the absence or presence of 5µM FGH10019 for 48 hours. All data are mean ± s.d. from n = 3 biological replicates.

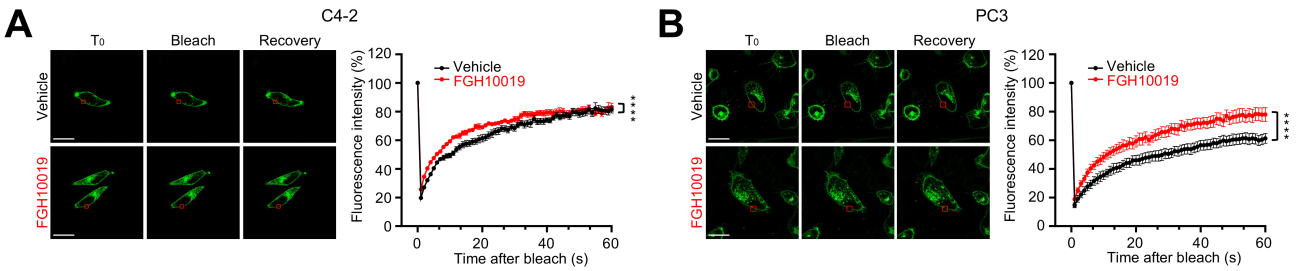

Fig. S2. Inhibition of SREBP-dependent lipogenesis increases cell membrane dynamics and the uptake of docetaxel. A, **B** Representative images and quantitation of FRAP analysis of C4-2 (**A**) or PC3 (**B**) cells treated with vehicle or 5μM FGH10019. Prostate cancer cells were transfected with a Lck-GFP plasmid for 24 hours and then treated with vehicle or 5μM FGH10019 for another 24 hours. Fluorescence was bleached in a specific region (red rectangle in **A**, **B**). Fluorescence recovery was then analyzed at the indicated time points. Scale bar, 20μm. Statistical significance was assessed using the extra sum-of-squares F test. ***$P < 0.001$. All data are mean ± s.d. from n = 3~5 biological replicates.

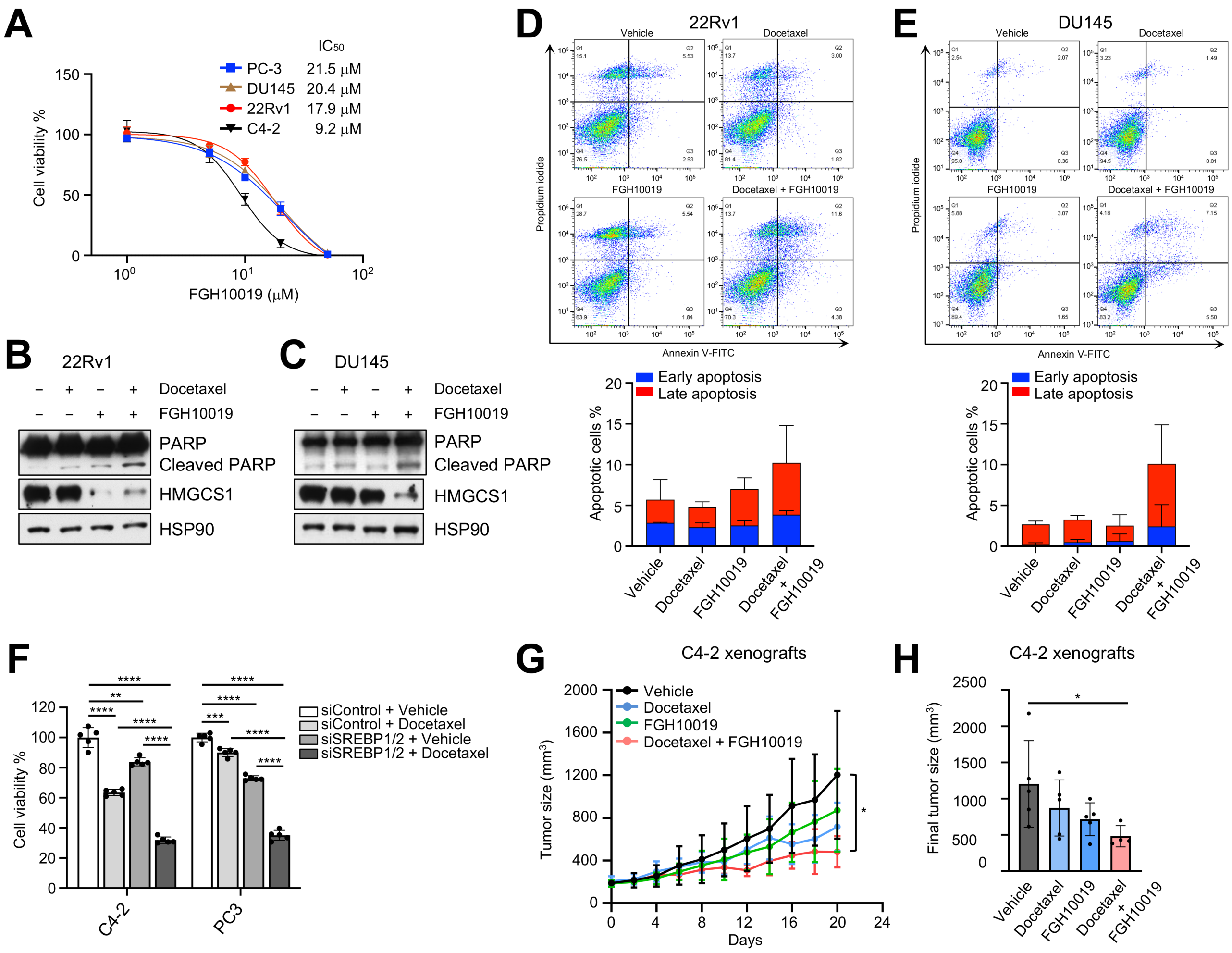

Fig. S3. Targeting of SREBP-dependent lipogenesis potentiates the antitumor activity of docetaxel in prostate cancer both *in vitro* and *in vivo*. A 72-hour dose response curves of FGH10019 treatment in various prostate cancer cell lines. **B**, **C** IB analysis of lysates from 22Rv1 (**B**) or DU145 (**C**) cell line treated with vehicle, 1nM docetaxel, 5μM FGH10019, or a combination of docetaxel and FGH10019 for 72 hours. **D**, **E** Representative FACS plots and quantitation of early and late apoptotic cells from 22Rv1 (**D**) or DU145 (**E**) cell line treated with vehicle, 1nM docetaxel, 5μM FGH10019, or a combination of docetaxel and FGH10019 for 72 hours. **F** Cell viability assay in C4-2 and PC3 cells. Prostate cancer cells were transfected with control or *SREBP1/2* siRNA mixtures for 24 hours, then treated with vehicle or 1nM docetaxel for 48 hours.

G, **H** Tumor volume over the course of treatment (**G**) or final tumor volume (**H**) of C4-2 xenografts after six-week treatment with vehicle, docetaxel, FGH10019, or a combination of docetaxel with FGH10019. n = 5 mice per group. Docetaxel was administered i.p. at a dose of 4 mg/kg body weight twice per week. FGH10019 was administered orally at a dose of 20 mg/kg body weight three times per week. In **F**, **H**, unpaired two-tailed *t* test was used to determine significance. In **G**, two-way ANOVA with Tukey's multiple-comparison test with Bonferroni's post hoc tests was used to determine significance. *$P < 0.05$, **$P < 0.01$, ***$P < 0.001$, ****$P < 0.0001$. All data are mean ± s.d.
